# The *Wolbachia* Genome of *Brugia malayi:* Endosymbiont Evolution within a Human Pathogenic Nematode

**DOI:** 10.1371/journal.pbio.0030121

**Published:** 2005-03-29

**Authors:** Jeremy Foster, Mehul Ganatra, Ibrahim Kamal, Jennifer Ware, Kira Makarova, Natalia Ivanova, Anamitra Bhattacharyya, Vinayak Kapatral, Sanjay Kumar, Janos Posfai, Tamas Vincze, Jessica Ingram, Laurie Moran, Alla Lapidus, Marina Omelchenko, Nikos Kyrpides, Elodie Ghedin, Shiliang Wang, Eugene Goltsman, Victor Joukov, Olga Ostrovskaya, Kiryl Tsukerman, Mikhail Mazur, Donald Comb, Eugene Koonin, Barton Slatko

**Affiliations:** **1**Molecular Parasitology Division, New England BiolabsBeverly, MassachusettsUnited States of America; **2**National Center for Biotechnology Information, National Library of MedicineNational Institutes of Health, Bethesda, MarylandUnited States of America; **3**Integrated Genomics, ChicagoIllinoisUnited States of America; **4**Parasite Genomics, Institute for Genomic ResearchRockville, MarylandUnited States of America; University of ArizonaUnited States of America

## Abstract

Complete genome DNA sequence and analysis is presented for *Wolbachia,* the obligate alpha-proteobacterial endosymbiont required for fertility and survival of the human filarial parasitic nematode *Brugia malayi.* Although, quantitatively, the genome is even more degraded than those of closely related *Rickettsia* species, *Wolbachia* has retained more intact metabolic pathways. The ability to provide riboflavin, flavin adenine dinucleotide, heme, and nucleotides is likely to be *Wolbachia*'s principal contribution to the mutualistic relationship, whereas the host nematode likely supplies amino acids required for *Wolbachia* growth. Genome comparison of the *Wolbachia* endosymbiont of B. malayi (*w*Bm) with the *Wolbachia* endosymbiont of Drosophila melanogaster (*w*Mel) shows that they share similar metabolic trends, although their genomes show a high degree of genome shuffling. In contrast to *w*Mel, *w*Bm contains no prophage and has a reduced level of repeated DNA. Both *Wolbachia* have lost a considerable number of membrane biogenesis genes that apparently make them unable to synthesize lipid A, the usual component of proteobacterial membranes. However, differences in their peptidoglycan structures may reflect the mutualistic lifestyle of *w*Bm in contrast to the parasitic lifestyle of *w*Mel. The smaller genome size of *w*Bm, relative to *w*Mel, may reflect the loss of genes required for infecting host cells and avoiding host defense systems. Analysis of this first sequenced endosymbiont genome from a filarial nematode provides insight into endosymbiont evolution and additionally provides new potential targets for elimination of cutaneous and lymphatic human filarial disease.

## Abstract 2

Analysis of this *Wolbachia* genome, which resides within filarial parasites, offers insight into endosymbiont evolution and the promise of new strategies for the elimination of human filarial disease

## Introduction

Over 1 billion people in more than 90 countries are at risk from filarial nematode infections, and 150 million people are infected. The parasitic nematodes are insect-borne and are responsible for lymphatic or cutaneous filariasis, leading to medical conditions including elephantiasis or onchocerciasis (African river blindness). Lymphatic filariasis is caused predominantly by Wuchereria bancrofti and Brugia malayi and affects 120 million individuals, a third of whom show disfigurement, while onchocerciasis, caused by *Onchocerca volvulus,* affects 18 million people of whom 500,000 have visual impairment and 270,000 are blind [1,2]. Within these filarial parasites are intracellular bacteria that were first observed almost 30 y ago [3,4,5,6].

The establishment in 1994 of a Filarial Genome Project funded by the World Health Organization (WHO/Tropical Disease Research/United Nations Development Programme/World Bank) contributed to the rediscovery of these endosymbiotic bacteria. In the analysis of cDNA libraries generated from different life cycle stages of *B. malayi,* the presence of rare non-*Escherichia-coli-*like, alpha-proteobacterial sequences implicated the occurrence of endobacterial DNA [7]. Phylogenetic analyses subsequently identified the bacteria as *Wolbachia* [8]. These endosymbionts have now been found in the vast majority of filarial nematode species, with notable exceptions [3,9,10,11,12,13,14,15,16,17,18,19]. *Wolbachia* appear to be absent in nonfilarial nematodes [20].

In nematodes that contain *Wolbachia* and which have been well examined, the bacteria are located in the lateral chords (invaginations of the body wall hypodermis that project into the body cavity) in both sexes. They are also localized in oocytes but not in the male reproductive tract. The endosymbionts appear to be present in 100% of individuals within a population, when that species contains them, suggesting that they are required for worm fertility and survival [10,21,22]. They are therefore potential therapeutic targets for filariasis control.

Certain antialpha proteobacterial agents, most notably tetracycline and doxycycline, but also rifampicin and azithromycin, show inhibitory effects on parasitic nematode development and fertility [13,23,24,25,26,27,28,29,30,31,32,33]. After antibiotic treatment, immunogold staining, using *Wolbachia-*specific cell-surface probes, shows the absence of *Wolbachia* in the female reproductive tract and the degeneration of embryos, while *Wolbachia* remain in the lateral chords, albeit in reduced numbers [34]. Genchi et al. [35] have also shown that *Wolbachia* are present at 1000X lower frequencies after antibiotic treatment and can still be detected by PCR from female hypodermis tissues, but not from female reproductive tissue. No antibiotic effects are observed in filarial nematodes that do not harbor *Wolbachia,* nor are they observed with other antibiotics (e.g., penicillin, gentamicin, ciprofloxacin, or erythromycin), suggesting that these effects correlate with *Wolbachia* presence [11,12,13,36,37]. Human trials using doxycycline, undertaken in Ghana, have shown that this antibiotic interferes with embryogenesis in adult female filariae with a concomitant depletion of *Wolbachia* from both adults and microfilariae (first stage larvae) of O. volvulus and W. bancrofti [38,39,40,41,42]. Thus, as in animal models, *Wolbachia* appears to be a therapeutic target for human filarial parasitic infections.

The use of anti-*Wolbachia* chemotherapy against filarial parasites has initiated a novel approach for filarial disease control and eradication. Previous strategies for elimination of filariasis have included vector control in the presence or absence of antiparasitic drugs [43,44,45,46,47]. Diethylcarbamazine, albendazole, and ivermectin have been the most recent drugs of choice for prevention of filarial infections, but they have little effect on adult worms. Thus repeated doses in endemic areas are required to eliminate infections that can arise again within months of treatment [39,44,48]. In addition, the possibility of drug resistance, as observed with intestinal helminths in animals is a concern [49,50,51]. No new therapeutics have been developed in over 20 y, and there is a need for better drugs that permanently sterilize or kill adult worms.

*Wolbachia* play a role in the host immunological response to filarial parasite invasion. Infection by filarial parasites results in B-cell proliferation and the generation of antibodies directed toward parasite- and *Wolbachia*-specific antigens, including those to *Wolbachia* surface protein, heat shock protein, aspartate aminotransferase, and Htr serine protease [11,52,53,54,55,56,57]. Other *Wolbachia*-specific molecules also play roles in the immune response to filarial infections including the release of stimulatory and modulatory factors from neutrophils and monocytes, which may be related to *Wolbachia* release upon worm death [58,59,60,61]. One component of the host immune response appears to mimic a lipopolysaccharide (LPS)-like response, typically observed as a host immune response to Gram-negative bacteria (such as the alpha-proteobacterial *Wolbachia*) [22,58,62,63,64,65]. Further, LPS-like products of *Wolbachia* appear to be involved in the eye inflammation observed in African river blindness. Leukocytes (neutrophils and eosinophils) infiltrate the cornea as a result of microfilarial invasion and death within the eye, leading to a loss of corneal transparency [66]. LPS-like molecules are implicated in this process due to activation of the toll-like receptor 4 (TLR4) pathway by *Wolbachia* [61,67].

Release of filarial worm-associated molecules, especially after drug treatments that cause worm death in the host, leads to pathogenesis (“Mazzotti Reaction”) [68,69,70,71,72], and *Wolbachia* has been associated with chronic and acute infection states of filariasis (reviewed in [59]). Repetitive exposures to LPS-like molecules due to release of *Wolbachia* following death of microfilaria are thought to induce chronic inflammation events giving rise to immune tolerance [65,73], as hyporesponsiveness occurs with increasing parasite load [74,75,76].

*Wolbachia* endosymbionts can be separated into six supergroups based upon 16S rRNA, *Wolbachia* surface protein, and *fts*Z phylogenetics [8,11,15,77,78,79,80,81,82]. Four supergroups contain *Wolbachia* from arthropods while supergroup C contains *Wolbachia* from the nematodes O. volvulus and *Dirofilaria immitis,* and supergroup D contains *Wolbachia* from *B. malayi, W. bancrofti,* and Litomosoides sigmodontis [11,82]. In nematodes, the evolution of *Wolbachia* parallels the phylogenetics of their hosts, while in the other supergroups, horizontal transmission appears to have occurred [11,14,15,79,82]. The closest bacterial relatives to the *Wolbachia* are in the Order Rickettsiales, including *Rickettsia, Ehrlichia, Cowdria,* and *Anaplasma,* all parasites of mammals that require arthropod vectors for transmission [83,84].

Up to 70% of all insect species appear to harbor *Wolbachia* [85,86,87]. While parasitic and maternally inherited in insects, they appear not to be required for host survival. But when present in appropriate genetic backgrounds, they confer developmental effects leading to sex ratio disturbances, feminization of genetic males, parthenogenesis, cytoplasmic incompatibilities and/or reciprocal-cross sterility [79,88,89,90]. It has been suggested that endosymbionts, including *Wolbachia,* might be of medical importance and used for insect vector control to deliver antiparasitic products to recipient hosts [91,92,93,94,95,96,97,98,99,100,101, 102]. For these reasons, a genome project was initiated and completed on the *Wolbachia* endosymbiont of Drosophila melanogaster (*w*Mel) [103].

Identification of *Wolbachia* in parasitic nematodes, their role in pathogenesis, their potential as a target for development of antifilarial therapeutics, and their widespread occurrence in arthropods triggered a meeting held in 1999 to initiate a consortium of *Wolbachia* researchers [104,105]. Three additional meetings have been held (see http://www.wolbachia.sols.uq.edu.au/index.html, and eight additional *Wolbachia* genomes responsible for diverse phenotypes are being sequenced.

We report the second complete genome sequence of *Wolbachia* and the first from a parasitic nematode, B. malayi (*W. pipientis,* BruMal TRS strain; *Wolbachia* endosymbiont of *B.* malayi [*w*Bm]). We also describe a comparative analysis of reductive evolution in different lineages of endosymbiotic bacteria, a major evolutionary trend in all intracellular parasites and symbionts. Features of the *w*Bm genome are presented as a systematic comparison to *w*Mel and *Rickettsia* spp., the closest fully sequenced relatives of *w*Bm and more distant intracellular parasites and symbionts of the gamma-proteobacterial lineage, such as *Buchnera* (aphid endosymbiont), *Blochmannia* (ant endosymbiont), and *Wigglesworthia* (tsetse fly endosymbiont) [106,107,108,109,110,111,112]. We also delineate the metabolic pathways that might account for the mutualistic relationship between *Wolbachia* and its nematode host.

## Results/Discussion

### Genome Properties and General Comparison with the Genomes of Other Parasites and Endosymbionts

The genome of *w*Bm is represented by a single circular chromosome consisting of 1,080,084 nucleotides and is 34% G+C. The size agrees with the 1.1 Mb length previously determined by both pulsed-field gel electrophoresis and restriction mapping [113,114]. The origin of replication *(oriC)* was tentatively mapped immediately upstream of the *hem*E gene on the basis of GC- and AT-skew analyses [115] (Figure 1). The genome of *w*Bm has an extremely low density of predicted functional genes compared to all other bacteria, with the exceptions of R. prowazekii (Table 1) and *Mycobacterium leprae.* Both *Wolbachia* spp. and *Rickettsia* spp. have undergone considerable gene loss in many metabolic pathways, relative to other alpha-proteobacteria (Table 2). A comparison of predicted functional genes in *w*Bm and *Rickettsia* spp. reveals a large core set that is conserved among these genomes, as well as smaller sets unique to each genome (Figure 2). In contrast, nearly all observed pseudogenes are unique to each genome (Figure 2), suggesting substantial independent genome degradation. *Wolbachia* (*w*Bm) and R. conorii contain, in addition to many demonstrable pseudogenes, a considerable number of short open reading frames (ORFs), which have no detectable orthologs in current protein databases but are recognized as probable genes by gene prediction programs. However, most of these sequences, which comprise approximately 5% of the total predicted gene number in *w*Bm, are likely to be fragmented genes as well (Table 1).

**Figure 1:**
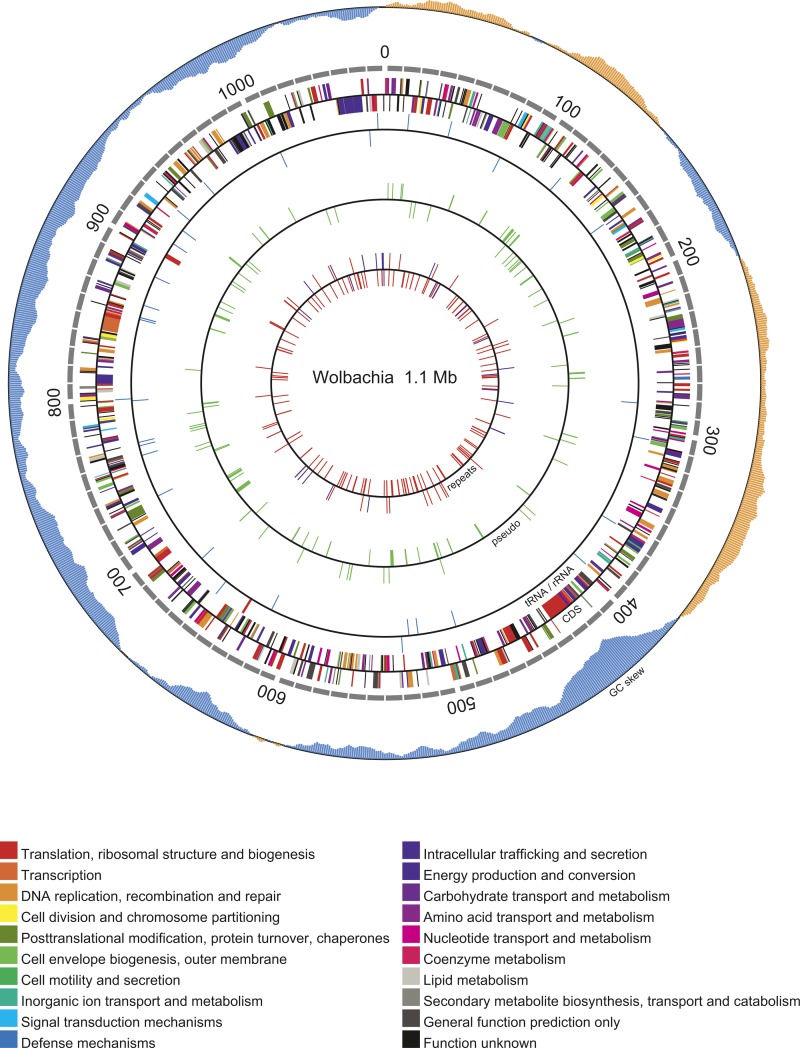
Genogram of the Complete Circular Genome of *w*Bm The scale indicates coordinates in kilobase pairs (kbp) with the putative origin of replication positioned at 0 kbp. The outermost ring indicates the GC-skew over all bases in the forward strand using a window size of 40 kbp and a step size of 1 kbp. Positive and negative skew are shaded gold and blue, respectively. Features are shown as paired rings separated by a circular baseline. In each pair, the outer and inner rings represent the forward and reverse DNA strands, respectively. Working inward from the scale, the features displayed are as follows: identified genes and their broad functional classification (multihued, as listed); tRNA (blue)/rRNA (red) genes; putative pseudogenes (green); repeated sequences (red) and transposon-related repeats (blue).

**Figure 2:**
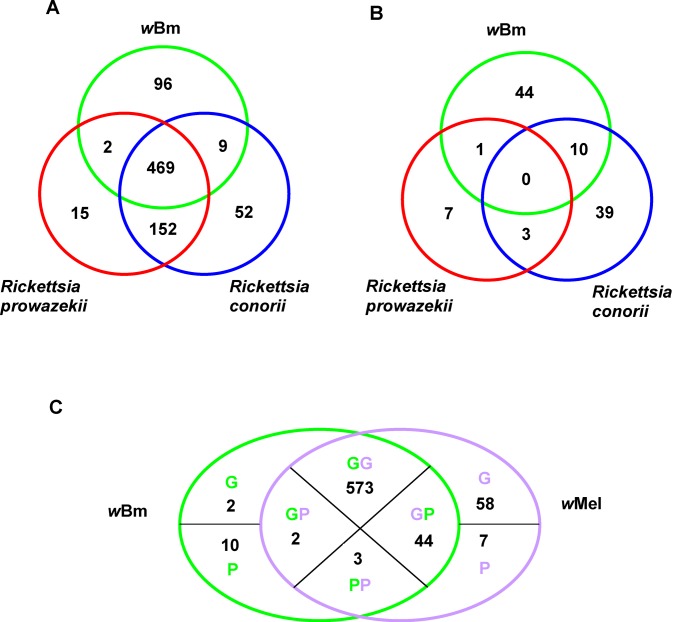
Venn Diagram Showing Comparison of Conserved and Unique Genes and Pseudogenes in *w*Bm *(Wolbachia* from *B. malayi), Rickettsia prowazekii, Rickettsia conorii,* and in *w*Bm and *w*Mel (among Those Assigned to COGs) (A) Predicted functional protein-coding genes. (B) Pseudogenes. (C) Combined results for comparison between *wBm* and *w*Mel. G, intact gene; P, pseudogene.

**Table 1:**
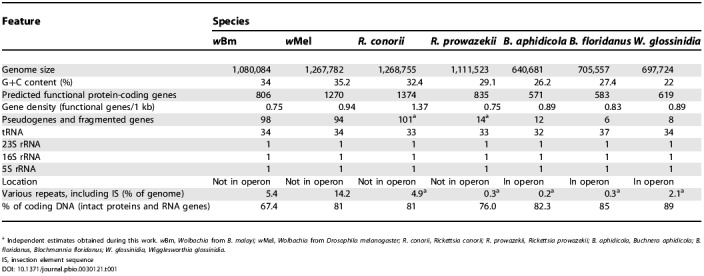
Comparison of Genome Features of Proteobacterial Endosymbionts and Endoparasites

^a^ Independent estimates obtained during this work. *w*Bm, *Wolbachia* from *B. malayi; w*Mel, *Wolbachia* from *Drosophila melanogaster; R. conorii, Rickettsia conorii; R. prowazekii, Rickettsia prowazekii; B. aphidicola, Buchnera aphidicola; B. floridanus, Blochmannia floridanus; W. glossinidia, Wigglesworthia glossinidia.*

IS, insection element sequence

**Table 2:**
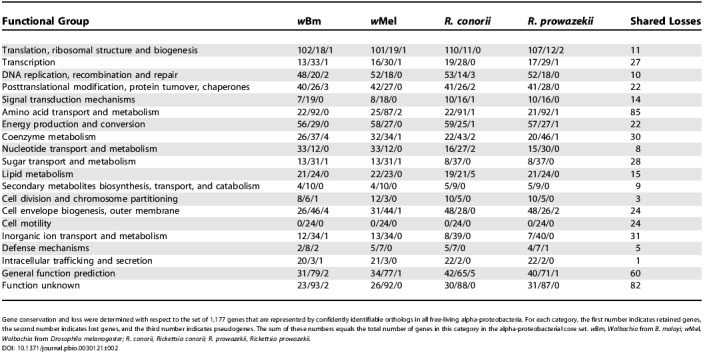
Gene Loss and Decay in *Wolbachia* and *Rickettsia*

Gene conservation and loss were determined with respect to the set of 1,177 genes that are represented by confidently identifiable orthologs in all free-living alpha-proteobacteria. For each category, the first number indicates retained genes, the second number indicates lost genes, and the third number indicates pseudogenes. The sum of these numbers equals the total number of genes in this category in the alpha-proteobacterial core set. *w*Bm, *Wolbachia* from *B. malayi; w*Mel, *Wolbachia* from *Drosophila melanogaster; R. conorii, Rickettsia conorii; R. prowazekii, Rickettsia prowazekii.*

The *w*Bm genome contains one copy of each of the ribosomal RNA genes (16S, 23S, and 5S), which do not form an operon, as also observed in *w*Mel and *Rickettsia* but in contrast to most other bacteria, and 34 tRNA genes that include cognates for all amino acids. Probable biological function was assigned to 558 (approximately 70%) of the 806 protein coding genes; a more general prediction of biochemical function was made for an additional 49 ORFs. Most of the predicted genes (617, 76%) could be included in clusters of orthologous groups of proteins (COGs) with orthologs not only in *w*Mel and *Rickettsia* but also in more distant organisms.

A lack of flagellar, fimbrial or pili genes indicates that *w*Bm is probably nonmotile (Table 2). However, some intracellular pathogens, including spotted fever group *Rickettsia,* exploit a different motility mechanism that makes use of the host cell actin polymerization to promote bacterial locomotion. Actin-based motility of *Rickettsia* depends upon activation of the host Arp2/3 complex by the WASP family protein RickA [116,117]. A gene coding for WASP family protein (Wbm0076) was identified in *w*Bm suggesting that it might be able to employ actin polymerization for locomotion and cell-to-cell spread.

### Informational and Regulatory Systems

Comparison with an obligatory gene set characteristic for free-living alpha-proteobacteria (Table 2) shows that both *Wolbachia* spp. and *Rickettsia* have retained an almost intact gene set for translational processes (greater than 84%). Several RNA metabolism genes are among the few shared losses, including tRNA and rRNA modification enzymes (LasT, RsmC, Sun, TrmA, CspR) and even pseudouridine synthase, TruB (pseudogenes in both lineages). TruB is present in all gamma-proteobacterial endosymbionts but absent in other parasites and endosymbionts, including *Mycoplasma, Chlamydia,* and spirochetes. It is likely that the lack of these modifications affects reading frame maintenance and translation efficiency in both *Wolbachia* spp. and in *Rickettsia.* Further reduction of genes involved in RNA modification occurs specifically in *w*Bm and in *w*Mel, which have lost several genes involved in queuosine biosynthesis (COG0809, COG603, COG702, COG0602, COG0780) [118] and 16S rRNA uridine-516 pseudouridylate synthase. The absence of RNA methylase (COG1189) highlights the loss of RNA modification systems, which is a general trend in evolution of endosymbionts among various lineages [119].

Although *w*Bm retains most of the genes for DNA replication and repair, the loss of several genes present in other alpha-proteobacteria (except *w*Mel) is notable. These include the chi subunit of DNA polymerase III (HolC), chromosome partitioning proteins ParB and ParA, repair ATPase (RecN), exonuclease VII (XseAB), and the RNA processing enzyme RNase PH (Rph).

Both *Wolbachia* spp. and *Rickettsia* have a complete repertoire of UV-excision (UVR-ABCD-mediated), recombinational synaptic (RecA/RecFOR-mediated), and postsynaptic (RuvABC-mediated) DNA repair pathways. In contrast, *Buchnera* and *Blochmannia* are devoid of conventional homologous recombination and uvr pathways, although they encode a putative phrB familyphotolyase [107,109,110,112,120,121]. *Wolbachia, Rickettsia, Buchnera,* and *Wigglesworthia* all encode enzymatic machinery to counter the deleterious effects of various types of base oxidative damage, which could be important for defense against mutagenic metabolic by-products in the intracellular environment [103,108,109,119,122].

Many proteins categorized as being involved in protein fate in the two *Wolbachia* spp. and *Rickettsia* spp. (CcmF, CcmB, CcmH, CcmE, CcmC, Cox11, CtaA), but which are absent in the genomes of gamma-proteobacterial endosymbionts, are involved in biogenesis of cytochrome c oxidase and c-type cytochromes typical of alpha-proteobacterial aerobic respiratory chains. Respiratory chains of gamma-proteobacterial endosymbionts employ quinol oxidase rather than cytochrome c oxidase.

A major loss of transcriptional regulators likely occurred in the common ancestor of *Wolbachia* and *Rickettsia* spp. (Table 2). Only a few of these genes have been additionally lost in the *w*Bm lineage, including those from COG1396, COG1959, COG1329, COG1678, and COG1475. This is a general trend in evolution of endosymbionts and parasites [118,122,123], suggesting that most of their genes are likely constitutively expressed. Those few regulators found in *w*Bm that are not present in other alpha-proteobacteria, including two Xre-like regulators (COG5606), may be of interest for future experimental characterization. Similarly, most genes implicated in signal transduction systems are absent in both *Wolbachia* and *Rickettsia* spp. Several regulatory proteins that remain in the genome are involved in various stress responses (Wbm0660, MerR/SoxR family; Wbm0707, cold shock protein; Wbm0494, stress response morphogen; Wbm0061, TypA-like GTPase) or in cell cycle regulation (Wbm0184, PleD-like regulator; Wbm0596, cell cycle transcriptional regulator CtrA).

### Metabolic Capabilities of *w*Bm are Key to Understanding its Interaction with the Host

One of the roles of *w*Bm as an obligate endosymbiont may be to provide its host with essential metabolites. Although *w*Bm has retained more metabolic genes than *Rickettsia* spp., its biosynthetic capabilities appear to be rather limited. Unlike *Buchnera* spp. [107,109,112,122,123], *w*Bm is able to make only one amino acid—*meso*-diaminopimelate (*meso*-DAP), a major peptidoglycan constituent. In most bacteria, it is produced as an intermediate in the pathway of lysine biosynthesis. Similar to *Rickettsia* spp. [122], *w*Bm lacks *meso*-DAP decarboxylase (LysA, COG0019), necessary for lysine biosynthesis, such that the biochemical pathway ends with *meso*-DAP.

Complete pathways for de novo biosynthesis of purines and pyrimidines are found in *w*Bm, as opposed to *Rickettsia* and many other endosymbionts and parasites, including *Buchnera, Blochmannia, Mycoplasma,* and *Chlamydia* (Table 3). The general trend for nucleotide biosynthesis pathways to be lost in these organisms appears to be independent of the presence of ADP/ATP translocase (COG3202) (present only in *Rickettsia* and *Chlamydia*), which facilitates the uptake of nucleotide-triphosphates from the hosts. This observation suggests that *w*Bm produces nucleotides not only for internal consumption but also for supplementation of the nucleotide pool of the host (Figure 3) when needed, such as during oogenesis and embryogenesis, where the requirement for DNA synthesis is likely very high [124].

**Figure 3:**
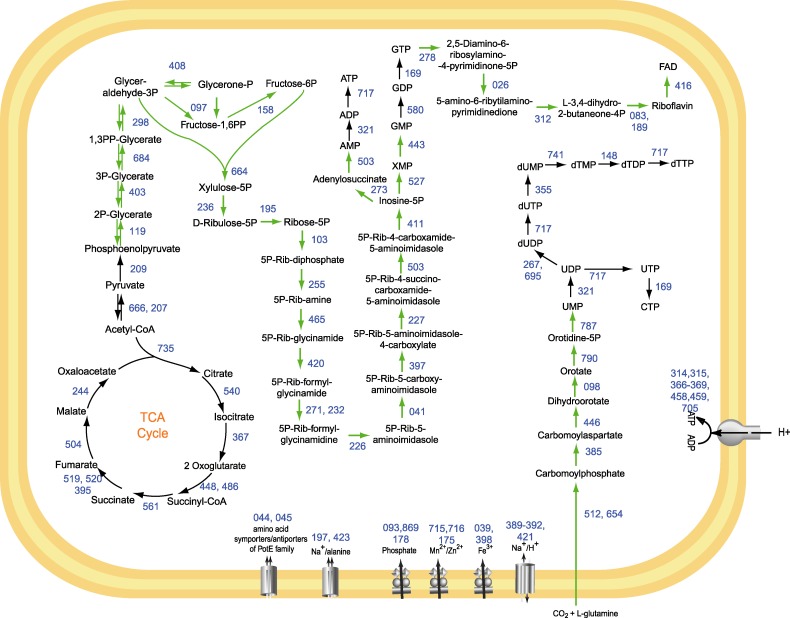
Metabolic Pathways Retained in wBm Pathways shared by *Wolbachia* and *Rickettsia* are shown with black arrows. Pathways present in *Wolbachia* but not in *Rickettsia* are shown with green arrows. Numbering alongside pathway arrows reflects enzyme annotation, a table of which is available at http://tools.neb.com/wolbachia/.

**Table 3:**
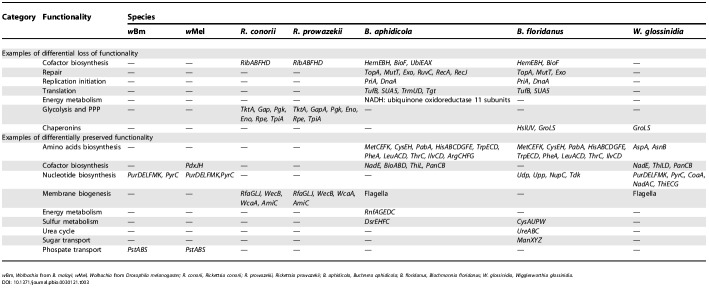
Differential Loss of Functionality and Differentially Preserved Functionality, if Only a Few Compared Alpha- and Gamma-Proteobacterial Parasite/Symbiont Genomes Have Lost or Preserved This Functionality

*w*Bm, *Wolbachia* from B. malayi; *w*Mel, *Wolbachia* from *Drosophila melanogaster; R. conorii, Rickettsia conorii; R. prowazekii, Rickettsia prowazekii; B. aphidicola, Buchnera aphidicola; B. floridanus, Blochmannia floridanus; W. glossinidia, Wigglesworthia glossinidia.*

All genes required for biosynthesis of fatty acids and all but one gene for biosynthesis of phospholipids (phosphatidylglycerol, phosphatidylserine, and phosphatidylethanolamine) are present in the *w*Bm genome. The absent gene in phospholipid biosynthesis is glycerol-3-phosphate acyltransferase (COG2937), which catalyzes the transfer of the first fatty acid to glycerol-3-phosphate. However, a “fatty acid/phospholipid biosynthesis enzyme” PlsX is present, which can complement the absence of glycerol-3-phosphate acyltransferase in E. coli [125]. All but one gene for biosynthesis of isoprenoids has been found in the genome. This absent gene is 1-deoxy-D-xylulose-5-phosphate synthase (COG1154), an essential gene in the nonmevalonate pathway. It is possible that this biochemical function could be complemented by a transketolase or transaldolase, two highly promiscuous enzymes encoded by the *w*Bm genome or, alternatively, 1-deoxy-D-xylulose-5-phosphate must be supplied by the host.

Unlike *Rickettsia, w*Bm contains all the enzymes for the biosynthesis of riboflavin and flavin adenine dinucleotide (Figure 3). *w*Bm could be an important source of these essential coenzymes for the host nematode. No genes for riboflavin biosynthesis have been detected in the ongoing B. malayi genome data (9X coverage) [126]. Similar to most other endosymbionts, *w*Bm lacks complete pathways for de novo biosynthesis of other vitamins and cofactors such as Coenzyme A, NAD, biotin, lipoic acid, ubiquinone, folate, and pyridoxal phosphate, retaining only a few genes for the finals steps in some of these pathways. These incomplete pathways may make *w*Bm dependent upon the supply of those precursors from the host.

Heme serves as a prosthetic group of cytochromes, catalase and peroxidase, and may be another metabolite provided by *w*Bm to *B. malayi. w*Bm has all but one gene for heme biosynthesis and has maintained all genes for maturation of c-type cytochromes. The absent gene in the heme biosynthesis pathway encodes protoporphyrinogen oxidase, a gene not identified in many alpha-proteobacteria. It is likely that these bacteria contain a functional form of protoporphyrinogen oxidase, which is not yet known, or that the missing function is complemented by another gene function, as in E. coli [127].

Heme could play an important role in filarial reproduction and development. It is possible that molting and reproduction are regulated by ecdysteroid-like hormones, since the insect hormones ecdysone and 20-hydroxyecdysone and their inhibitors affect molting and microfilarial release in D. immitis and B. pahangi [128,129]. In *Drosophila,* five enzymatic reactions in the pathway of ecdysteroid biosynthesis are catalyzed by microsomal and mitochondrial cytochrome P450 mono-oxygenases [130]. If similar enzymes participate in the pathway of biosynthesis of filarial steroid hormones, heme depletion caused by elimination of *w*Bm could result in a decreased activity of these enzymes, which might account for the effects on nematode viability, larval development, and reproductive output observed following antibiotic treatment of filarial parasites.

There is currently no evidence of heme biosynthesis enzymes in B. malayi (analysis of the draft genome sequence of B. malayi does not identify any genes for heme biosynthesis [126]). These enzymatic activities have been detected in *Setaria digitata,* a cattle filarial parasite, which is devoid of typical cytochrome systems, yet has heme-containing enzymes, such as microsomal cytochrome P450, catalase, and peroxidase [131]. It is not known whether S. digitata contains *Wolbachia* and whether heme biosynthesis detected in this worm is due to the presence of endosymbiotic bacteria. However the closely related filarial parasites, *S. equina, S. tundra,* and S. labiatopapillosa are devoid of endosymbiotic *Wolbachia* [15,16]; perhaps they have retained the genes for heme biosynthesis.

Genes for biosynthesis of glutathione are present in the *w*Bm genome (Wbm0556; Wbm0721). Two physiological roles of glutathione in bacteria are known: one is detoxification of methylglyoxal [132], and the other is protection against oxidative stress through activation of the glutathione peroxidase–glutathione reductase system [133,134]. Methylglyoxal is accumulated in phosphate-limited environments, such as those encountered by *Salmonella* inside macrophages [132]. It is possible that *w*Bm encounters phosphate-limited conditions inside the host and therefore needs glutathione as a quencher of methylglyoxal. This view is supported by the presence of the gene encoding the Kef-type potassium efflux system, a participant in methylglyoxal detoxification through acidification of cytosol [132]. However, no homologs of *E. coli glo*A–*glo*B genes responsible for glutathione-dependent methylglyoxal detoxification were found in the genome. Glutathione peroxidase is also absent, hence the physiological role of glutathione in *w*Bm is unclear. Although genes for glutathione biosynthesis are present in the B. malayi genome, it is possible that *w*Bm provides glutathione to the host, since the latter needs high levels of this essential metabolite for protection against oxidative stress [135] and detoxification [136].

Intermediates for these biosynthetic pathways are likely derived from gluconeogenesis, the nonoxidative pentose phosphate shunt, and the tricarboxylic acid (TCA) cycle. Glycolytic enzymes encoded by *w*Bm probably function in a gluconeogenesis pathway (Figure 3), since the genes coding for two enzymes catalyzing irreversible glycolytic reactions, 6-phosphofructokinase and pyruvate kinase, are absent. Instead, the gluconeogenic enzyme fructose-1,6-bisphosphatase (Wbm0132) and pyruvate-phosphate dikinase (Wbm0209), which functions predominantly in gluconeogenesis in bacteria, are present suggesting that the pathway functions as gluconeogenesis, albeit ending with fructose-6-phosphate rather than glucose-6-phosphate. While fructose-6-phosphate is necessary for biosynthesis of the peptidoglycan components N-acetylglucosamine and N-acetylmuramate, no enzymes capable of utilizing glucose-6-phosphate as a substrate are encoded in the *w*Bm genome.

It is reasonable to suggest that the most likely growth substrates for *w*Bm would be those compounds that are highly abundant in the worm. In adult *B. malayi, B. pahangi,* and *Dipetalonema viteae (Acanthocheilonema viteae),* these include the excretory metabolites lactate and succinate, which are the principal products of glucose utilization under both aerobic and anaerobic conditions, and a disaccharide trehalose, which is used by the worms as a storage compound [137,138]. Nuclear magnetic resonance studies of adult B. malayi identified phosphoenolpyruvate as the major energy reservoir [139]. However, *w*Bm is not predicted to be able to utilize lactate due to the absence of genes coding for lactate dehydrogenases and is likely unable to grow on sugars, as evidenced by the lack of genes encoding sugar transporters or sugar kinases. Thus, the most likely growth substrates for *w*Bm are pyruvate and TCA cycle intermediates derived from amino acids, with enzymes present for amino acid degradation, a pyruvate dehydrogenase complex, a complete TCA cycle, and a respiratory chain typical of alpha-proteobacteria (Figure 3). Amino acids are likely imported from the extracellular environment where they are obtained by proteolysis of host proteins by proteases and peptidases. Indeed, the genome of *w*Bm encodes a variety of proteases, including predicted metallopeptidases (at least seven Zn-dependent proteases of four distinct families compared to only one in *Rickettsia*) (Wbm0055, Wbm0153, Wbm0221, Wbm0311, Wbm0419, Wbm0418, Wbm0742). In addition, two Na^+^/alanine symporters were found (Wbm0197, Wbm0424), which are absent in *Rickettsia.*

### Cell Wall Structure

A dramatic case of lineage-specific gene loss in both *Wolbachia* spp. includes approximately 20 genes for enzymes of cell-envelope LPS biosynthesis. It has been reported that soluble endotoxin-like products of *Wolbachia* endosymbionts of filarial nematodes, including *B. malayi, B. pahangi, L. sigmodontis, O. volvulus,* and *D. immitis,* contribute to the immunology and pathogenesis of filarial diseases through induction of potent inflammatory responses, including production of tumor necrosis factor alpha, interleukin-1-beta, and nitric oxide by macrophages [22,58,59,60,71,72,140,141]. Chemokine and cytokine responses to the sterile extracts of *Brugia* and *Onchocerca* were dependent on signaling through TLR4 and could be blocked by neutralizing antibodies to CD14 and by the antagonistic lipid A analogs, indicating that the inflammatory response was induced by an LPS-like molecule. Recently the major surface protein of *Wolbachia* spp. was implicated as the inducer of the immune response acting in a TLR2- and TLR4-dependent manner [141]. However, it is not clear whether this protein is the only *Wolbachia*-specific molecule eliciting a TLR4-dependent innate immune response.

Analysis of the *w*Bm genome indicates that, like Ehrlichia chaffeensis and Anaplasma phagocytophilum [142], it lacks homologs of the genes responsible for biosynthesis of lipid A. Although lipid A structure can vary in different bacteria, it always consists of a polysaccharide backbone carrying fatty acid residues. The only predicted genes belonging to the glycosyltransferase family were those participating in peptidoglycan biosynthesis, and one glycosyltransferase pseudogene is present. Similarly, the only genes from the acyltransferase family are those participating in fatty acid and phospholipid biosynthesis. Thus, it is unlikely that the cell wall of *w*Bm contains LPS-like molecules. This idea is supported by the absence of the gene products responsible for maintaining the outer membrane structure in Gram-negative bacteria, such as TolQ, TolR, TolA, and TolB.

Several lines of evidence suggest that the structure of the *w*Bm peptidoglycan is very unusual, and peptidoglycan derivatives might be responsible in part for the observed inflammatory responses. First, although all the genes necessary for biosynthesis of lipid II are present in the *w*Bm genome, there are no homologs of alanine and glutamate racemases responsible for synthesis of pentapeptide components D-alanine and D-glutamate. While the genomes of *Rickettsia* spp. contain L-alanine racemase that could catalyze racemization of both alanine and glutamate, the only amino acid racemase present in the genomes of both *Wolbachia* is *meso*-DAP epimerase (Wbm0518), an enzyme catalyzing interconversions of LL- and *meso-*isomers of diaminopimelate. It is possible that *meso*-DAP epimerase is able to catalyze racemization of alanine and glutamate, although this activity has never been experimentally demonstrated. Alternatively, instead of the usual D-isomers, *w*Bm peptidoglycan might contain L-isomers of alanine and glutamate.

Second, Gram-negative bacteria (including *Rickettsia* spp.) usually contain two monofunctional transpeptidases. One of them, FtsI (also known as PBP3), is localized to the septal ring and is required for peptidoglycan biosynthesis in the division septum, while the other, PBP2, is localized preferentially to the lateral cell wall [143]. FtsI and PBP2 are recruited to the sites of their action by two membrane proteins, FtsW and RodA, respectively. In the *w*Bm genome, only functional orthologs of E. coli RodA and PBP2 were found; the orthologs of FtsW–FtsI are disrupted by multiple frameshifts.

Third, genomes of bacteria that have peptidoglycan in their cell wall usually contain at least one gene coding for a high molecular weight penicillin-binding protein responsible for cross-linking of the murein sacculus. The transpeptidase and transglycosylase domains of this protein catalyze transpeptidation and transglycosylation of the murein precursors, respectively, to form the carbohydrate backbone of murein and the interstrand peptide linkages. No homologs of bifunctional transpeptidase/transglycosylase or monofunctional biosynthetic transglycosylase were found in the genomes of *Wolbachia* spp., although they are present in the *Rickettsial* genomes. The homolog of lytic transglycosylase, which is responsible for hydrolysis of the carbohydrate backbone during bacterial growth and division, is also absent from the genomes of both *Wolbachia* spp. Thus, their peptidoglycan can be cross-linked by the interstrand peptide linkages, but the carbohydrate backbone is not polymerized. These observations suggest that peptidoglycan of *w*Bm has some features in common with the peptidoglycan-derived cytotoxin produced by Neisseria gonorrhoeae and Bordetella pertussis [144,145] and that muramyl peptides derived from *w*Bm peptidoglycan could elicit the inflammatory response contributing to the pathogenesis of filarial infection.

### Other Host Interaction Systems

As expected, functional Type IV secretion genes were found in the *w*Bm genome, including two operons: Wbm0793–Wbm0798 and Wbm0279–Wbm0283. These systems are indispensable for successful persistence of endosymbionts within their hosts [146]. Similar genes have been observed in the sequence of *w*Mel [103].

A role in the adaptation to the intracellular existence seems likely for several genes that are present in *w*Bm, *w*Mel, and *Rickettsia.* Thus, *w*Bm encodes five ankyrin-repeat-containing proteins and, in addition, has at least seven related pseudogenes, while *w*Mel contains 23 ankyrin -repeat-containing genes. *Rickettsia* contains two or three functional ankyrin-repeat genes (and probably one pseudogene) [147]. In eukaryotes, ankyrins connect cell membranes, including membranes of endosymbionts to the cytoskeleton [148], while in bacteria the function of ankyrin-like proteins remains largely unknown. One physiological function of bacterial ankyrin-like proteins was demonstrated in *Pseudomonas aeruginosa,* where ankyrin repeat AnkB is essential for optimal activity of periplasmic catalase, probably serving as a protective scaffold in the periplasm [149]. Another ankyrin-repeat protein, AnkA from *E. phagocytophila,* was detected in association with chromatin in infected cells, suggesting its possible role in regulation of host cell gene expression [150].

Another interesting protein is a member of the WASP family and is conserved in *Rickettsia* and *w*Bm (Wbm0076). Eukaryotic homologs of these proteins are suppressors of the cAMP receptor and regulate the formation of actin filaments [151]. The genes for an ankyrin-repeat protein and a WASP protein might have been acquired from a eukaryotic host by the common ancestor of *Rickettsia* and *Wolbachia* and could have contributed to the evolution of the intracellular lifestyle of these bacteria. *w*Bm also encodes several proteins with large nonglobular or transmembrane regions or internal repeats, orthologs of which are present also in the *w*Mel genome (Wbm0010, Wbm0304, Wbm0362, Wbm0749, and others). These proteins are likely to be surface proteins interacting with host cell structures.

### Further Comparisons of *w*Bm and *w*Mel

One of the most striking characteristics of the *w*Mel genome is a large amount of repetitive DNA and mobile genetic elements, including three prophages, altogether comprising more that 14% of genomic DNA (and about 134 ORFs). Despite the abundance of repeats in the *w*Bm genome (5.4%) (Figure 4), the percentage of repetitive DNA in *w*Bm is considerably less than in *w*Mel. This may reflect a stronger selection in *w*Bm for repeat loss and, as no prophages were identified in the *w*Bm genome, little exposure to foreign DNA. No plasmid maintenance genes were identified in the *w*Bm genome.

**Figure 4:**
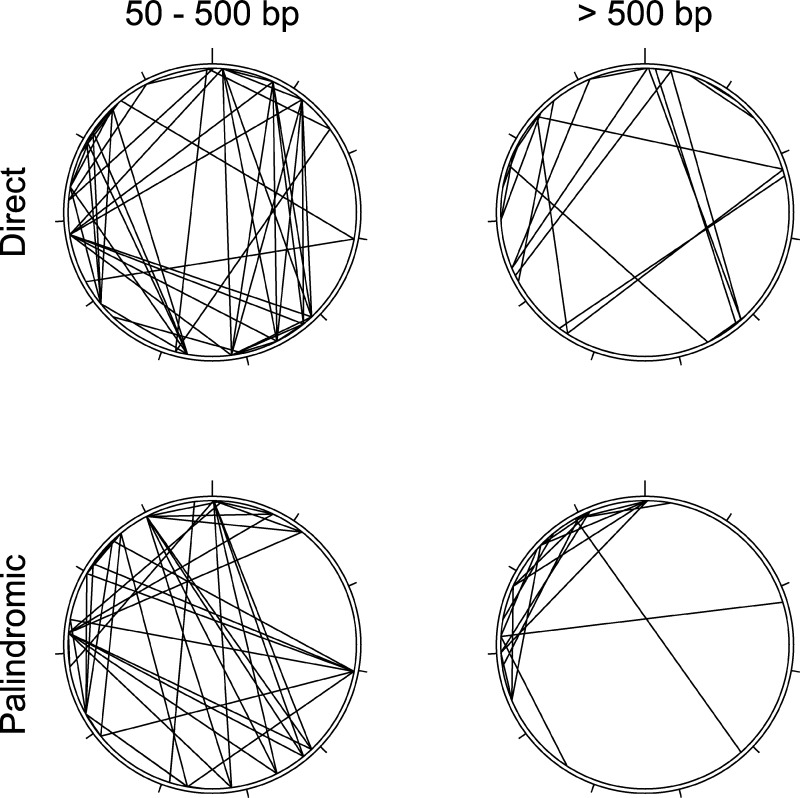
Organization of Direct and Palindromic Repeats in *w*Bm Circles represent the complete genomic sequence of *w*Bm. Repeats were identified using the REPuter program [182] and are connected by line segments. Direct repeats are shown in the graphs in the top row, while palindromic repeats are shown in the lower row of graphs. The left column graphs display repeats of 50 to 500 bp in length. The rightmost graphs display repeats of greater than 500 bp in length.

Comparison of the repetitive elements between these two genomes suggests the invasion of mobile genetic elements occurred after the divergence of the two *Wolbachia* along the *w*Mel branch, or that the majority of the transposons and phages were eliminated (degraded) specifically in the *w*Bm lineage. There is a similarly large difference in the amount of repetitive DNA in the two *Rickettsia* species (Table 1). While an appropriate outgroup would be useful in both comparisons, the apparent degradation of repetitive DNA in *Buchnera* spp. [111,112,152,153,154,155] suggests the specific elimination of nonessential DNA is a result of reduced selection on gene functions no longer necessary in the host cells in *Wolbachia* spp. [156]. The large number of repeats and an apparently active system of DNA recombination suggest that extensive genome shuffling within *w*Bm and *w*Mel has eliminated colinearity between their genomes (Figure 5). Frequent rearrangements in *Wolbachia* might be expected, given the exceptionally high levels of repeated DNA and mobile elements and the presence of several prophages in *w*Mel. It has been suggested that the surprisingly high percentage of repetitive DNA in *w*Mel might reflect a lack of selection for its elimination [103]. An alternative hypothesis might be that in *Wolbachia* there is a selective benefit to systems that maintain genetic diversity and that a high percentage of repeats may contribute to genome plasticity, as has been suggested for *Helicobacter* [157]. It has been suggested that the presence of a high level of repetitive DNA in *w*Mel, relative to *w*Bm, might reflect recurrent exposures to mobile elements and bacteriophages, as a result of its parasitic lifestyle [156,158].

**Figure 5:**
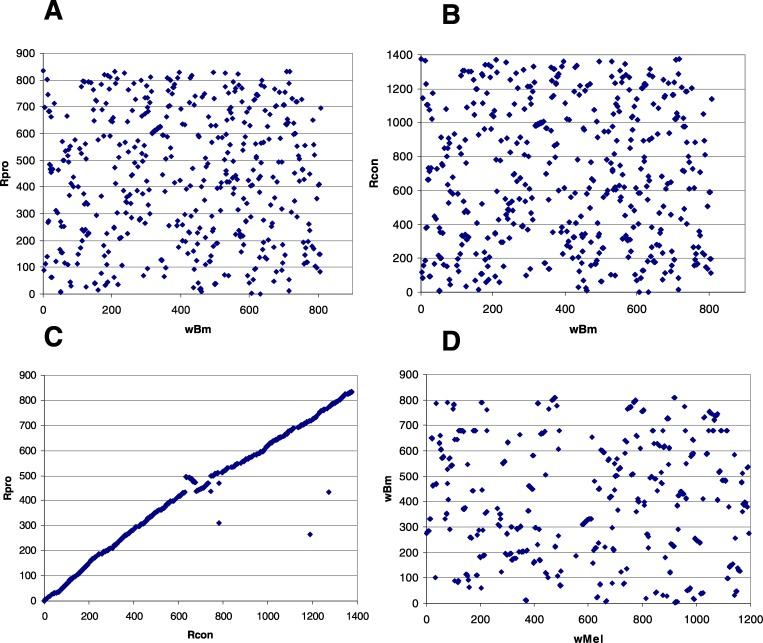
Absence of Gene Order Colinearity between *w*Bm and *Rickettsia* and Disruption of Gene Colinearity between *w*Bm and *w*Mel Each dot represents a pair of probable orthologs defined as reciprocal BLAST best hits with E-value less than 0.001. (A) Genome dot-plot comparison of *w*Bm *(Wolbachia* from *B. malayi)* and *Rpro (R. prowazekii).* (B) Genome dot-plot comparison of *w*Bm *(Wolbachia* from *B. malayi)* and *Rcon (R. conorii).* (C) Genome dot-plot comparison of *Rpro (R. prowazekii)* and *Rcon (R. conorii).* (D) Genome dot-plot comparison of *w*Bm and *w*Mel.

Comparative analysis of the genes assigned to COGs in both *w*Mel and *w*Bm shows that the genome of *w*Bm is more reduced (Figure 2; Table 2). In total, 696 individual proteins from wBm have an ortholog in the *w*Mel genome; 84 such proteins are not assigned to COGs, and a considerable fraction of them are specific for only these two genomes. At least half of these predicted genes are larger than 100 amino acids, and orthologs have a similar length and presumably encode functional proteins. One of the important differences between the two *Wolbachia* for which genomes are available is that *w*Bm is apparently a mutualistic symbiont of its host, while *w*Mel is parasitic. The smaller size of the *w*Bm genome might be related to this difference. *w*Mel likely has to retain genes required for infecting host cells and avoiding host defense systems, whereas wBm may have lost many of these genes, as has been seen in organelles and other mutualistic symbionts such as the *Buchnera* symbionts of aphids.

Despite there being considerably fewer predicted genes in *w*Bm (Table 1), the metabolic capabilities of *w*Mel and *w*Bm are very similar. Unlike *w*Bm, *w*Mel has retained some enzymes for folate and pyridoxal phosphate biosynthesis, two subunits of cytochrome bd-type quinol oxidase, and a few additional enzymes for amino acid utilization (proline dehydrogenase and threonine aldolase). Among the genes unique to *w*Bm, there are two extracellular metallo-peptidases (Wbm0384, Wbm0742) that are only distantly related to counterparts in the *w*Mel genome. These results suggest a basic common strategy used by *w*Bm and *w*Mel during the evolution of their host symbiosis. In the case of *w*Bm, the basis of the interaction may be to provide essential vitamin cofactors, heme biosynthesis intermediates, and nucleotides while requiring amino acids and perhaps other nutrients supplied by the host.

Both *Wolbachia* have lost a considerable number of membrane biogenesis genes that make them apparently unable to synthesize lipid A, the usual component of proteobacterial membranes. However, a few differences do exist. For example, in *w*Mel there is a predicted gene belonging to the family of GDSL-like lipases (WD1297), similar to the major secreted phospholipase of Legionella pneumophila [159], which also has phospholipid-cholesterol acyltransferase activity. Its ortholog in *w*Bm is disrupted by a frameshift (Wbm0354 corresponds to the C-terminal portion of the gene). However, it is still possible that, similar to E. chaffeensis and A. phagocytophilum [160], *w*Bm and *w*Mel incorporate cholesterol into their cell walls. Furthermore, *w*Mel retains several genes absent in *w*Bm that might be involved in cell wall biosynthesis. These include a small gene cluster (WD0611–WD0613) and several other enzymes (WD0620, WD0133, WD0431), suggesting that *w*Mel might produce peptidoglycan modified with an oligosaccharide chain, while *w*Bm makes unmodified peptidoglycan. Possible differences in peptidoglycan structure may be additionally predicted by the already mentioned loss of FtsW–FtsI genes in *w*Bm and their presence in *w*Mel. These differences may reflect the occurrence of a mutualistic lifestyle (*w*Bm) in contrast to a parasitic lifestyle (*w*Mel).

Somewhat surprisingly, no recent apparent horizontally transferred genes from hosts were found in either *Wolbachia* genome. Moreover, an aforementioned WASP protein homolog, apparently acquired by a common ancestor of *Wolbachia* and *Rickettsia* from an animal host, is disrupted in the *w*Mel genome (WD0811). However, in *w*Mel there are two proteins encoded in the region of the prophages (WD0443, WD0633) that have “eukaryotic” OTU-like protease domains with their predicted catalytic residues apparently intact [161]. Proteases from this family are shown to be involved in ubiquitin pathways [162]. To our knowledge, this is a rare appearance of these proteases in prokaryotic genomes, although they are present in the genomes of C. pneumoniae [161] and in a closely related genome, Chlamydophila caviae (CCA00261).

### Conclusions

Comparing the genomes of *w*Bm and *Rickettsia* to those of gamma-proteobacterial symbionts points to general similarities and distinctions in the evolution of endosymbionts. The genomes of R. conorii and *Wolbachia* species contain numerous repeats of various classes that are much more abundant than in the gamma-proteobacterial endosymbionts (Table 1). This correlates with the minimal gene colinearity between the genomes of *Wolbachia* and *Rickettsia* [103,114,163] (Figure 5). By contrast, gamma-proteobacterial endosymbionts share a variety of operons with one another, and even with free-living relatives, despite the dramatic gene loss. Furthermore, gamma-proteobacterial endosymbionts (with the exception of *Wigglesworthia*) have lost crucial genes involved in recombinational repair, whereas almost no gene loss in this functional class was observed in *Wolbachia* or *Rickettsia* spp. Active recombination between repeats might have led to both gene loss and genome shuffling in *Wolbachia* and *Rickettsia* spp., whereas other mechanisms of genome reduction were probably involved in the evolution of gamma-proteobacterial endosymbionts [109,120,121,122,123,164]

Comparative genome analysis highlights the different metabolic capabilities that render endosymbionts indispensable to their hosts [108,119,121]. For example, *Buchnera* and *Blochmannia* retain a nearly complete repertoire of amino acid biosynthesis pathways and supply amino acids to their insect hosts [110,112]. In contrast, *w*Bm, *w*Mel, and *Wigglesworthia* [103,108] have lost nearly all of these pathways but retain the pathways for the biosynthesis of nucleotides and some coenzymes (Table 3). Thus, endosymbiotic organisms in different divisions of proteobacteria independently evolved distinct strategies for symbiont–host interactions.

Genomic analysis of the alpha-proteobacterium *w*Bm, the first sequenced endosymbiont from a human parasitic nematode, provides new insights into the evolution of intracellular bacterial symbiosis and clues to the role of *Wolbachia* in the mutualistic relationship with the nematode. It is anticipated that continued genome analysis of nematodes and their endosymbionts will provide novel targets for antimicrobials aimed at the elimination of human filarial parasites.

## Materials and Methods

### Materials and Methods

B. malayi microfilaria worms were purchased from TRS Labs (Athens, GA, United States) for preparation of DNA. Because of the difficulties in obtaining purified *Wolbachia* DNA from the B. malayi host, bacterial artificial chromosome (BAC) libraries were created [114]. From these libraries, a minimum tiling path of 21 *Wolbachia* BACs was created and used for subcloning into plasmid vectors for genomic sequencing. This ordered BAC approach was useful in the assembly phase of the project because of the highly repetitive nature of this genome.

For plasmid library generation, equal amounts of BAC DNAs were pooled and 50 μg of DNA from the pool was sheared into 2.0–3.0 kb fragments (HydroShear device, GeneMachines, Genomic Solutions, Ann Arbor, Michigan, United States). Sheared DNA was purified from a 0.7% agarose gel, blunted, and cloned into cleaved, dephosphorylated plasmid vectors. Libraries were generated containing DNA from 1 to 9 BACs.

Plasmid DNA was isolated by a modified alkaline lysis protocol. Sequencing reactions were performed at Integrated Genomics (Chicago, Illinois, United States) using the DYEnamic ET Dye Terminator Cycle Sequencing Kit (Amersham Biosciences, Little Chalfont, United Kingdom). Unincorporated dye was removed by isopropanol precipitation as recommended by the manufacturer. Samples were run on MegaBace 1000 (Amersham Biosciences) sequencers; 87% of plasmid sequencing reactions were successful. The genome was sequenced to an average coverage of 10.7X and at 2X minimum coverage (at least once in each direction) and assembled.

The sequence was assembled into contigs by using PHRED–PHRAP–CONSED [165,166,167], and gaps were initially closed by primer walking (1,766 reactions). Regions considered to be potential frame shifts or sequencing errors after the first round of annotation were resequenced from direct genomic PCR products. The completed sequence was used to identify homologous sequences in the independent ongoing B. malayi sequence project (TIGR parasites genome database: http://www.tigr.org/tdb/e2k1/bma1/ [126]). The sequence of one BAC had been previously determined [163]. The final assembly was in full agreement with the BAC physical map [114].

Integrated Genomics ERGO software [168] and other software programs [169] were used for ORF calling, gene identification, and feature recognition. Computational analysis of the genome sequence was performed as previously described. Briefly, the tRNA genes were identified using the tRNA-SCAN program [170], and the rRNA genes were identified using the BLASTN program [171]. For the identification of the protein-coding genes, the genome sequence was conceptually translated in six frames to generate potential protein products of ORFs longer than 100 codons. These potential protein sequences were compared to the database of proteins from the COG database using COGNITOR [172].

After manual verification of the COG assignments, the validated COG members from *w*Bm were called as protein-coding genes. The COG assignment procedure was repeated with ORFs of greater than 60 codons from the intergenic regions. Additionally, the potential protein sequences were compared to the nonredundant protein sequence database using the BLASTP program [171] and to a six-frame translation of unfinished microbial genomes using the TBLASTN program [171], and those sequences that produced hits with E (expectation) values less than 0.01 were added to the protein set after an examination of the alignments. Finally, protein-coding regions were predicted using the GeneMarkS program [173]. After manual refinement, the genes predicted with these methods in the regions between evolutionarily conserved genes were added to produce the final protein set. Protein function prediction was based primarily on the COG assignments. In addition, searches for conserved domains were performed using the Conserved Domain Database (CDD) search option of BLAST (http://www.ncbi.nlm.nih.gov/Structure/cdd/wrpsb.cgi) and the SMART system [174], and in-depth, iterative database searches were performed using thePSI-BLAST program [175]. The KEGG database [176] (http://www.genome.ad.jp/kegg/metabolism.html) and the Integrated Genomics ERGO database pathway collection [168] were used, in addition to the COGs, for the reconstruction of metabolic pathways. Paralogous protein families were identified by single-linkage clustering after comparing the predicted protein set to itself using the BLASTP program [171]. Signal peptides in proteins were predicted using the SignalP program [177], and transmembrane helices were predicted using the MEMSAT program [178]. Gene orders in bacterial genomes were compared using the Lamarck program [179].

Two closely related genome sequences were completed and published since the above comparative analysis was undertaken [180,181].

## Supporting Information

### Data Access

DNA sequence, ORF, as well as annotation and positional information tables, are available at the following Web site: http://tools.neb.com/wolbachia/.

### Accession Number

The genome sequence was deposited in GenBank (http://www.ncbi.nlm.nih.gov/) under accession number AE017321.

## Abbreviations

BAC: bacterial artificial chromosome
COGs: clusters of orthologous groups of proteins
kbp: kilobasepairs
LPS: lipopolysaccharide
*meso*-DAP: *meso*-diaminopimelate
ORF: open reading frame
TCA: tricarboxylic acid
TLR4: toll-like receptor 4
*w*Bm: *Wolbachia* endosymbiont of *Brugia malayi*
*w*Mel: *Wolbachia* endosymbiont of Drosophila melanogaster
